# Genomic Basis of Adaptive Divergence in Leg Length between Ground- and Tree-Dwelling Species within a Bird Family

**DOI:** 10.1093/gbe/evad166

**Published:** 2023-09-14

**Authors:** Miaomiao Huang, Yanrui Liu, Xin Lu

**Affiliations:** Department of Ecology, College of Life Sciences, Wuhan University, Wuhan, China; Department of Ecology, College of Life Sciences, Wuhan University, Wuhan, China; Department of Ecology, College of Life Sciences, Wuhan University, Wuhan, China

**Keywords:** adaptive evolution, comparative genomics, leg-dependent locomotion, Paridae birds, *PTPA*

## Abstract

Hind limbs of tetrapods vary greatly in length and the variability can be associated with locomotor adaptation. Although the phenotypic evolution has been well documented, the underlying genetic basis remains poorly understood. We address this issue by integrating comparative genomics and functional prediction with a study system consisting of ground-dwelling, long-legged and tree-dwelling, short-legged species within the avian family Paridae. Genome-wide divergence and phenotypic correlation analyses jointly identified five highly divergent genomic regions that are significantly related with the difference in leg length between these two groups. Gene annotation for these regions detected three genes involved in skeletal development, that is, *PTPA*, *BRINP1*, and *MIGA2*, with the first one being under the strongest selection. Furthermore, four single nucleotide polymorphisms (SNPs) in the coding region of *PTPA* can well distinguish the two groups with distinct leg length. Among the four SNPs, one is non-synonymous mutation, and according to the prediction for protein structure and function, it can affect the 3D structure of the encoded protein by altering the corresponding amino acid's position. The alleles of *PTPA* were found in all sequenced species of the orders Palaeognathae and Psittaciformes, which typically take a ground locomotion style. A whole-genome scanning across bird species uncovered that the four SNPs are more likely to be present in resident passerines with increased leg length/wing length ratios (a proxy of leg-dependent locomotion efficiency). Our findings provide insight into the molecular evolution of locomotion performance based on leg morphology in birds.

**Significance**

Variation in leg length of tetrapods is well known to be associated with locomotor lifestyle. However, genetic mechanisms underlying this association are not yet fully understood. Here, we fill this gap using genomic data from an avian family that contains both ground-dwelling and tree-dwelling species, which differ obviously in leg length. Our study suggests that the gene *PTPA* may be associated with the divergence in leg length between the two avian groups as well as others with distinct locomotor lifestyle depending on leg length.

## Introduction

Throughout the history of animal evolution, a striking phenomenon is that the successful adaptation of locomotor organs can promote the development of body structure and function (Beckett 1986). This is because locomotion plays a crucial role in determining the ability of animals to find food and escape predators, which requires coordination of other organ systems. In tetrapods, hind limbs are the major drivers of moving on the ground or other substrates (Abourachid and Hofling 2012). This is particularly true in birds, where forelimbs have been specialized as wings. Moreover, within an animal group including birds, leg-dependent locomotor ability varies greatly in response to specific lifestyles and environments. As a result, leg morphology and function diverge substantially among species. In birds, for example, ostriches and jungle fowls are adept at walking on the ground relying on their longer or strong legs; in contrast, swifts that spent most of their life in the sky have shorter legs that facilitate grasping cliffs.

Although the phenotypic adaptation of leg morphology has been well known, there is a need to explore molecular mechanisms behind the adaptation. In this regard, the existing work has primarily focused on the complete degeneration or intense modification of limbs in snakes (Coates and Ruta 2000; Greene and Cundall 2000; Kvon et al. 2016) and cetaceans (Gingerich et al. 1990; Thewissen and Fish 1997; Thewissen et al. 2006), with an emphasis on loss of gene function. However, a more common phenomenon occurs where leg morphology varies progressively and genetic mechanisms behind the variability should be due to allelic differentiation of the genes related to leg morphology between taxa with different locomotor performances. To our knowledge, only a few studies reported genetic changes associated with leg elongation in mammals (Cretekos et al. 2008; Osterwalder et al. 2018; Chavez et al. 2022). Birds demonstrate a remarkable case of extensive adaptive radiation within many taxonomic groups (Cooney et al. 2017), which accordingly produces divergence in locomotor performance and leg morphology. Therefore, these taxonomic groups provide an excellent opportunity to explore the genomic basis of adaptive leg morphology.

**Fig. 1.**
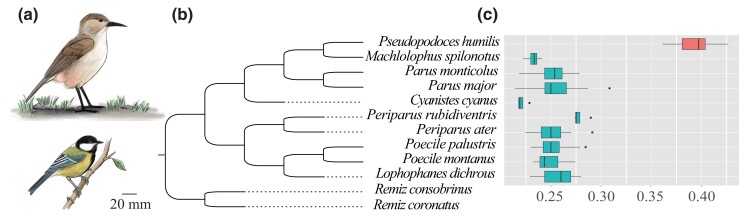
An interspecific comparison of leg length relative to wing length among Paridae species used in this study. (a) Tibetan ground tits and great tits with distinct locomotion lifestyles, (b) phylogenetic tree, and (c) leg length/wing length. Box plots represent interquartile range and the median and whiskers 1.5 times the vertical box boundaries.

Here, we address this issue using species in the family Paridae as a model system. All but 1 of the 64 Paridae species are adapted to tree-dwelling, spending most daytime moving between branches and roosting in trees overnight. The only exception to the tree-dwelling lifestyle is the Tibetan ground tit (*Pseudopodoces humilis*) endemic to the Tibetan plateau (Johansson et al. 2013). The phylogenetic tree based on 68 single-copy orthologs suggests that the ground tit diverged from the yellow-cheeked tit (*Machlolophus spilonotus*) about 7.7 Mya (Qu et al. 2013). Ground tits inhabit treeless, open meadows of 2,500 to 6,000 m elevation (Lu et al. 2011a; Lu et al. 2011b), where they spend almost daytime in ground movements characterized by hopping on the ground, feeding on soil invertebrates, nesting, and roosting in 80–290 cm long burrows under the ground, which are excavated by themselves using their curved bill and powerful legs (Ke and Lu 2009; Wang and Lu 2011). In response to the ground-dwelling lifestyle, ground tits have evolved longer, stronger legs as compared with their congeners (fig. 1).

Based on whole-genome resequencing data, we investigated the genetic basis of adaptive variation in leg length of the Tibetan ground tit versus its congeners (table 1). Specifically, we tried to find the genes that are primarily responsible for variation in leg length between the two groups (long-legged ground tits and its short-legged congeners). Perhaps one might argue that the difference in leg length between the two groups could only represent a taxonomic rather than adaptive trait to locomotion. To rule out the possibility, we searched for alleles of the genes associated with variation in leg length of the Paridae species among the whole genomes of 536 bird species in relation to leg-dependent locomotion efficiency.

**Table 1:** Species Used in This Study

Scientific Name	Common Name	No. of Sequenced Individuals	Lifestyle
Paridae species			
*P. humilis*	Ground tit	20	Ground-dwelling
*Cyanistes cyanus*	Azure tit	3	Tree-dwelling
*Lophophanes dichrous*	Grey-crested tit	9	Tree-dwelling
*M. spilonotus*	Yellow-cheeked tit	5	Tree-dwelling
*Parus major*	Great tit	6	Tree-dwelling
*Parus monticolus*	Green-backed tit	4	Tree-dwelling
*Periparus ater*	Coal tit	8	Tree-dwelling
*Periparus rubidiventris*	Rufous-vented tit	10	Tree-dwelling
*Poecile montanus*	Willow tit	6	Tree-dwelling
*Poecile palustris*	Marsh tit	6	Tree-dwelling
Outgroup			
*Remiz consobrinus*	Chinese penduline tit	2	
*Remiz coronatus*	White-crowned penduline tit	2	

## Results

### Genetic Regions Associated with Leg Length

**Fig. 2.**
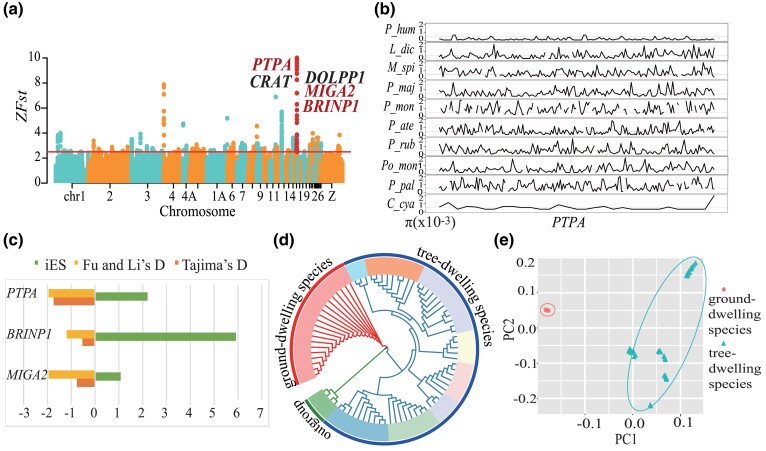
Multiple neutrality tests. (a) Manhattan plot of genome-wide F_ST_ analysis between the ground-dwelling, long-legged group and tree-dwelling, short-legged group. Red lines represent cutoff values of 2.5 in Z*F*_ST_ analysis. Candidate genes of bone development are in red. (b) Nucleotide diversity (π) of *PTPA*, with the red line being 0.005 of *π*. (c) Selection pressure analysis for three candidate genes within Tibetan ground tits. (d) Phylogenetic tree based on *PTPA* coding region SNPs. (e) PCA for coding region SNPs.

Based on standardized *F*_ST_ analyses and the Partial Mantel Test (PMT) (with the criteria of Z*F*_ST_ > 2.5, correlation coefficients *r* > 0.5, and *P* < 0.05), we identified five genomic regions that stand out as the outliers between long-legged ground tits and their short-legged congeners (table 2; supplementary fig. S1, Supplementary Material online). Through single nucleotide polymorphism (SNP) annotation, a total of five genes are present in these regions, including BMP/retinoic acid inducible neural specific 1 (*BRINP1*), mitoguardin 2 (*MIGA2*), carnitine *O*-acetyltransferase (*CRAT*), dolichyldiphosphatase 1 (*DOLPP1*), and protein phosphatase 2 phosphatase activator (*PTPA*) (fig. 2*a*).

**Table 2:** Five Windows under Divergent Selection Identified by *F*_ST_ Analyses and the Partial Mantel Test. Genes Related to Bone Development Are Bolded

Windows	*Genes*	*F*_ST_	Z*F*_ST_	*R*	*P*
chr17:6515001-6565001	Intergene region	0.560	7.191	0.582	0.049
chr17:6590001-6640001	Intergene region	0.486	3.244	0.543	0.042
chr17:5415001-5465001	***PTPA***, *CRAT*, *DOLPP1*, ***MIGA2***	0.475	2.700	0.538	0.049
chr17:6855001-6905001	***BRINP1***	0.472	2.504	0.561	0.046
chr17:7250001-7300001	Intergene region	0.472	2.504	0.572	0.046

Gene function annotation and literature investigation show that three of the five genes are related to bone development. *BRINP1* operates by encoding retinoic acid, a determinant of the anterior–posterior polarity of legs in chickens and mice (Tabin 1991; Tickle and Eichele 1994) as well as facial morphological remodeling in chickens (Lee et al. 2001; Schneider et al. 2001). *MIGA2* is associated with a significant ontological term “bone development” (GO: 0060348), which implies that this gene takes part in the process of formation and maturation of bone structures. In addition, this gene has been demonstrated to play a role in the pathogenesis of mice osteoporosis (Bassett et al. 2012). *PTPA* can regulate osteoblast differentiation by encoding a specific phosphatase activator (Xie et al. 2020). It also participates in BMP-Smad signaling pathway, which is important in skeletal growth (Gazzerro and Canalis 2006; Bengtsson et al. 2009).

### Neutrality Tests for the Genes Related to Bone Development

For all the three genes found to be related with bone development, nucleotide diversity (*π*) is lower in ground tits than in its short-legged congeners (supplementary table S3, Supplementary Material online). In particular, *PTPA* has the lowest *π* value in ground tits (fig. 2*b*). Each of the three genes does not differ in the haplotype block length (fig. 2*c*). In ground tits, *PTPA* has a stronger signal of selection (Tajima's *D*, −1.73964; Fu and Li's *D*, −1.73964) than *BRINP1* (Tajima's *D*, −0.49974; Fu and Li's *D*, −1.18985) and *MIGA2* (Tajima's *D*: −0.76169; Fu and Li's *D*, −1.95388) compared with other Paridae species, as indicated by two neutrality tests (fig. 2*c*). Taken together, *PTPA* is most likely to be associated with the divergence in leg length between the two groups of species.

We also performed phylogenetic and principal component analyses (PCA) to determine which regions of *PTPA* are under selection. We found that the coding regions can typically separate ground tits as a monophyletic group from short-legged species (fig. 2*d*), but the intron regions fail to do so (supplementary fig. S2, Supplementary Material online). The PCA also demonstrated that SNPs located in the coding region can distinguish between ground tits and its congeners (fig. 2*e*).

### Mutation in *PTPA*'s Coding Region and Protein Structure

**Fig. 3.**
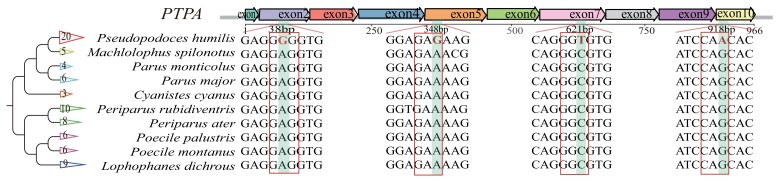
*PTPA* sequence alignment in ten Paridae species. The number in the front indicates how many samples have the allele.

By aligning the protein-coding sequences of *PTPA* in Paridae species, we found four SNPs that were able to tell ground tits from their short-legged congeners (fig. 3). They are 38 bp A/G in exon 2, 348 bp A/G in exon 5, 621 bp C/T in exon 7, and 918 bp G/A in exon 10. Of these four SNPs, only 38 bp A/G in exon 2 is a nonsynonymous substitution (E13G). We set PROVEAN confidence threshold at −2.5 to assess whether an amino acid substitution affects functions of the coded protein. We found that this mutation gives rise to a harmful variant protein (−2.9). According to the protein structure predicted by AlphaFold2, an alternation of glutamate (E) to glycine (G) occurs at position 13 (E13G), with a deviation from the original position at the N-terminal region (supplementary fig. S3, Supplementary Material online). It has been known that N-terminal processing is involved in post-translational modification in the expression of function of almost all proteins, including phosphorylation that is the main regulatory pathway of the *PTPA*.

### Potential Adaptive Substitution across the Avian Tree

**Fig. 4.**
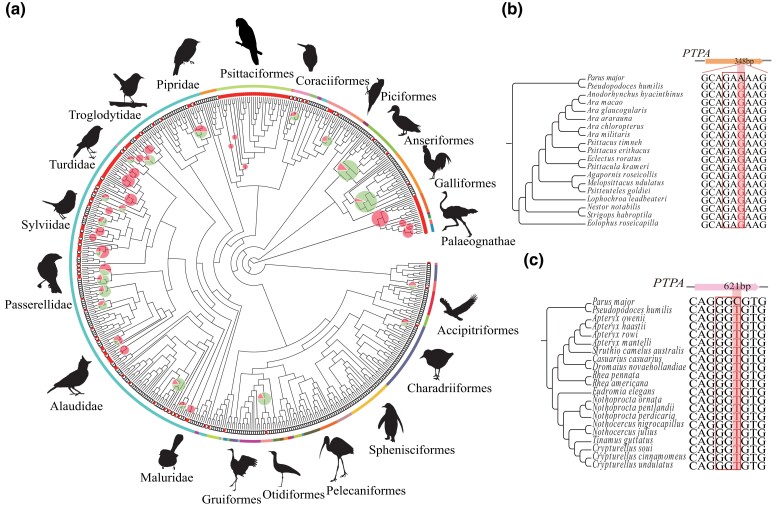
Scanning of the alleles of *PTPA* across the avian tree. (a) Distribution of the alleles in the phylogenic tree of 536 bird species (the bird silhouettes were obtained from phylopic.org). The outermost line denotes the species coverage of a specific taxonomic group. The red-filled circle indicates that at least one mutation is present in a species, and the white-filled circle indicates that the mutation is absent. The proportion of red circles in the branch indicates the percentage of family members with and without the mutation. (b) *PTPA* sequence alignment in parrots. (c) *PTPA* sequence alignment in ratites.

To further investigate whether *PTPA* involves divergence in leg morphology of other avian groups, we scanned the 4 alleles of *PTPA* across 536 species with known genome sequences (fig. 4*a*). Before doing so, we were sure that our reconstructed sequences are consistent with the published sequences in ground tits (XM_005527259.2) and great tits (XM_015644761.2). We detected the alleles in all sequenced species in the orders Psittaciformes (fig. 4*b*) and Palaeognathae (fig. 4*c*), which have much large leg length/wing length ratios and strongly depend on ground locomotion.

**Fig. 5.**
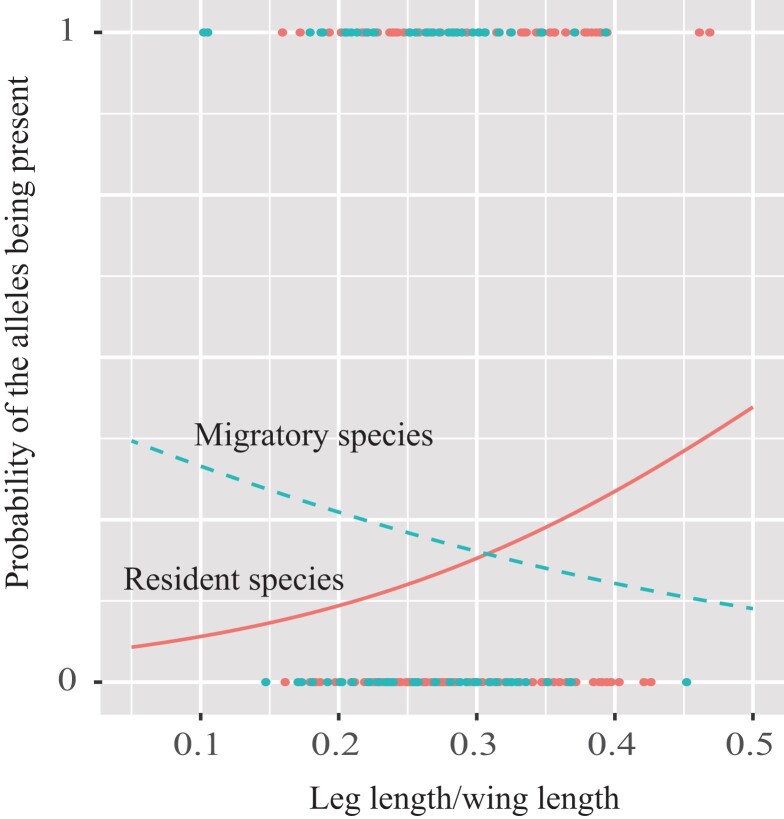
Leg length/wing length ratio as a predictor of the probability to detect the alleles of *PTPA* across 220 Passeriformes species, according to phylogenetic generalized linear mixed models. Dots are raw data, and lines show the predicted relationships for secondary species (red, with the solid line showing near significant) and migratory species (green, with the dotted line showing nonsignificant).

Phylogenetic generalized linear mixed model analyses revealed that the probability of the alleles of *PTPA* being present in a bird species is not predicted by leg length/wing length ratio, migratory status, and their interaction in both non-Passeriformes and combined with Passeriformes species (table 3). However, across Passeriformes species, the interaction between leg length/wing length ratio and migratory status is nearly significant, suggesting that in the order Passeriformes, the gene associated with leg length is more likely to be present in resident species that perform better in ground locomotion (fig. 5).

**Table 3:** The Results of Phylogenetic Generalized Linear Mixed Model Analyses Investigating Whether Leg Length/Wing Length Ratio Can Predict the Presence of the Alleles of *PTPA* across Bird Species. The Data Used in the Analyses Excluded 12 Species with Measures of Leg and Wing Length Unavailable, Three Species with Phylogenetic Information Unavailable, and Four Flightless Species in Non-Passeriformes

Models	*β*	SE	*Z*	*P*
All species (*n* = 517)				
Leg/wing ratio	0.9914	4.3525	0.2278	0.8198
Migratory status	−0.1555	0.9144	−0.1700	0.8650
Leg/wing ratio × migratory status	0.0606	3.5360	0.0171	0.9863
Non-Passeriformes species (*n* = 297)				
Leg/wing ratio	−0.7070	7.1594	−0.0988	0.9213
Migratory status	−1.4254	1.4403	−0.9897	0.3223
Leg/wing ratio × migratory status	1.2699	6.7619	0.1878	0.8510
Passeriformes species (*n* = 220)				
Leg/wing ratio	14.7777	7.7679	1.9024	0.0571
Migratory status	2.7958	1.5250	1.8333	0.0668
Leg/wing ratio × migratory status	−9.0922	5.4722	−1.6615	0.0966

## Discussion

This research aims to find molecular evidence for adaptive variation in leg length between a ground-dwelling bird and its tree-dwelling congeners in the avian family Paridae through scanning whole genomes. *PTPA* associated with bone development was considered to be a major gene that involves in the locomotion-related phenotypic adaptation. One of the four SNPs in this gene was found to alter the structure of the coded protein. In addition, for 536 bird species with known genome sequences, the alleles were more likely to be detected in those with a larger leg length/wing length ratio. To our knowledge, this is the first comparative genome approach to identify the genes underlying genetic divergence in locomotion ability based on leg morphology among birds.

Our findings are robust for three major reasons. First, we have identified the genomic regions that are potentially associated with leg length by using two methods, namely, Z*F*_ST_ and the PMT. Both approaches enable us to pinpoint the specific genomic regions that are most likely to be responsible for the observed variations in leg length between species with distinct lifestyles of locomotion. Moreover, all the three identified genes from the candidate regions have been reported to involve in bone development, suggesting that the regions we identified have a stronger signal associated with leg length in the genome than other regions. Second, multiple methods were employed to test for neutrality evolution, including analyses of genetic diversity, haplotype, and two *D*-values. We thus were able to identify gene *PTPA* that is subject to the strongest selection pressure among the three genes related to bone development. This gene can well distinguish the ground-dwelling species from its tree-dwelling congeners, especially at the four SNPs according to the phylogenetic tree and PCA analyses. Third, the selected alleles we identified from Paridae species are demonstrated to exist among two avian orders where the functions of leg-dependent locomotion are very strong. Furthermore, the analysis of functional morphology revealed a significant association of relatively long legs with ground locomotion across the avian tree (Xu et al. 2023).

We acknowledge that the current study is only the first step toward exploring the molecular mechanisms of locomotion adaptation based on leg morphology in birds. Future studies should make functional tests for the gene *PTPA* related to leg development. Insights into the genetic mechanisms also depend on further investigations on genes and regulatory pathways that directly determine the growth of long bone in birds and other animals. We detected that the probability to find the alleles of *PTPA* tends to increase with leg length/wing length ratio only among resident passerine birds. Nevertheless, this gene is absent in many taxa with long legs. It is likely that leg length does not always reflect the locomotion capacity of a species or taxonomic group and poor quality of genome. Instead, long legs may serve other functions such as increasing strides in cranes and storks (Navalon et al. 2022). We thus suggest that within-taxon approaches could have greater potential to capture the molecular mechanisms underlying leg-dependent locomotion than across-taxon approaches, as we have done for Paridae species. Therefore, we encourage more studies to explore this issue for more specific avian groups.

## Materials and Methods

### Whole-Genome Data

For Tibetan ground tits, the data of whole-genome resequencing were obtained through Illumina sequencing for 20 individuals. The Illumina sequencing procedure is briefly described below. Genomic DNA was extracted from an ethanol-preserved blood sample (∼50 µl) taken from the wing vein of birds captured in the field using DNeasy Blood kits. High-throughput resequencing was performed by taking an Illumina HiSeq X Ten instrument with paired-end length of 150 bp. We collected the whole-genome resequencing data of 9 tree-dwelling species (table 1) in the family Paridae (57 individuals) from the European Bioinformatics Institute (EMBL-EBI) (supplementary table S1, Supplementary Material online).

### SNP Calling

We processed the raw Illumina data with Trimmomatic v. 0.36 (Bolger et al. 2014) to pick out paired-end sequences. Low-quality bases were further removed from both ends of each read by running the adapter trimming using the same software with default parameters. FastQC (Andrews 2010) was employed to assess the efficacy of adapter trimming, which may ensure a high quality of the remaining bases. Using BWA v. 0.7.15 (Li and Durbin 2010), the resulting clean reads were mapped to the genome (GCA_ 001522545.3) of the great tit *Parus major*, a Paridae species that has the highest-quality reference genome available (Laine et al. 2016).

SNP polymorphisms were identified by performing Genome Analysis Toolkit (GATK) v. 3.8 (McKenna et al. 2010) HaplotypeCaller according to the Best Practices recommendations. To remove low-quality SNPs, the hard filtering parameters were set with a density distribution strategy provided by GATK: QD < 2.0 || MQ < 40.0 || FS > 60.0 || SOR > 3.0 || MQRankSum < −5.0 || ReadPosRankSum < −8.0. Clean SNPs were obtained by GATK with the default settings after removing duplicates.

### Genomic Regions Associated with Leg Length

To seek the genomic regions that exhibit a divergence between the ground- and tree-dwelling Paridae species, we used VCFtools v. 0.1.13 (Danecek et al. 2011) with nonoverlapping 50 kb windows (Cheng et al. 2020) to calculate the fixation index (*F*_ST_) between the two groups. The *F*_ST_ values were then Z-transformed (Z*F*_ST_) to eliminate the background genetic divergence. Considering that leg length is the most important trait associated with the two distinct locomotion lifestyles, we conducted the PMT (Mantel 1967), a procedure that can integrate the leg length matrixes and pairwise *F*_ST_ matrixes into a single analysis. These two matrixes were constructed using the *dist* package in R function (Ihaka and Gentleman 1996). The shared genomic regions identified by both methods can be considered to be the genetic signal associated with leg length.

### SNP Annotation

SNPs located within the shared regions were annotated by SnpEff v. 4.3 (Cingolani et al. 2012), and the annotation results were manually verified using Genome Data Viewer (https://www.ncbi.nlm.nih.gov/genome/gdv/browser/gene/? id=102111589). Prior to running the SnpEff, a local database was constructed based on the annotated genome of the great tits (GCA_ 001522545.3) deposited in the National Center for Biotechnology Information. Proofreading in Genome Data Viewer was carried out to ensure the accuracy of the annotation results. Enrichment analyses based on Gene Ontology for the annotated genes (supplementary table S2, Supplementary Material online) were performed with DAVID (Huang et al. 2009), utilizing well-annotated ontologies of chickens, mice, and humans.

### Selection Signal

We conducted a series of neutral statistical tests to identify the genes that have undergone divergent selection using the obtained SNP markers (supplementary table S3, Supplementary Material online). Nucleotide diversity (*π*) was calculated for all SNPs using VCFtools v. 0.1.13. Tajima's *D* (Tajima 1989) and Fu and Li's *D* (Fu and Li 1993) were calculated using DnaSP (Librado and Rozas 2009). The integrated extended haplotype homozygosity statistic (iES) was calculated in the *rehh* (Gautier and Vitalis 2012) package in R function to yield haplotype-based linkage disequilibrium (LD). Phylogenetic trees were reconstructed using PAUP (Swofford 1998) based on the SNPs from coding region and open reading frame regions, respectively, aiming to determine the molecular markers that were able to distinguish the ground-dwelling species from its tree-dwelling congeners. To verify the result, a PCA was performed using PLINK v. 1.9 (Purcell et al. 2007).

### Mutations in Candidate Genes Related to Leg Length

To identify mutations and predict functions of the candidate genes, we reconstructed coding sequences (CDS) of these genes from the SNP data. We also retrieved the CDS for ground tits and great tits from NCBI to validate the sequences’ accuracy. The CDS were aligned using MEGA-X software (Kumar et al. 2018) between all species to identify SNPs that can distinguish the ground-dwelling species from its tree-dwelling congeners. If non-synonymous mutations were detected, the structure and function of the coded proteins were predicted with AlphaFold2 (Jumper et al. 2021) and PROVEN software (Choi and Chan 2015), respectively.

### Whole-Genome Scanning across the Avian Tree for SNPs Related to Leg Length Identified in the Paridae Species

To see whether the SNPs identified to be related to variation in leg length among Paridae species are commonly present in birds, we scanned the whole genomes of 536 bird species from NCBI (supplementary table S4, Supplementary Material online). Before scanning, the local Blast library was constructed. The great tit's sequences were used to query other species with the reciprocal TBlast (Altschul et al. 1990), setting the *E* value thresholds according to the best-hit method as 1 × 10^−5^. The sequence with the highest match was retained, and the entire CDS was presented using an in-house script. Subsequently, we aligned the CDS and searched for these alleles across these bird species.

Moreover, we employed phylogenetic generalized linear mixed model analyses to examine whether the probability to detect the SNPs can be predicted by leg-dependent locomotion capacity in birds. The locomotion capacity was estimated with the ratio of leg length/wing length. Data on the two morphological parameters were taken from AVONET (Tobias et al. 2022). A set of 1,000 phylogenetic trees of 524 species with whole-genomic data available were download from BirdTree (www.birdtree.org) (Jetz et al. 2012), and these trees were summarized into a consensus tree using the maximum clade credibility method (Heled and Bouckaert 2013). When fitting the phylogenetic generalized linear mixed model, the link function was set as logit and the error distribution as binomial. All statistical analyses were performed using the R software (R Core Team 2013).

## Supplementary Material

evad166_Supplementary_Data

## Data Availability

Illumina sequencing data for ground tits can be found on NCBI at PRJNA998938.
