# Supplementary material for: Genomic Basis of Adaptive Divergence in Leg Length between Ground- and Tree-Dwelling Species within a Bird Family
Source: Genome Biol Evol. 2023 Sep 14;15(9):evad166. doi: 10.1093/gbe/evad166 (PMC10516731; doi:10.1093/gbe/evad166)

**Genomic basis of adaptive divergence in leg length between ground- and tree-dwelling species within a bird family**

**Supplementary Material**

Miaomiao Huang, Yanrui Liu, Xin Lu

Department of Ecology, College of Life Sciences, Wuhan University, Wuhan, 430072, China

Email: [xlu@whu.edu.cn](mailto:xlu@whu.edu.cn)

This file includes:

Figures S1 to S3

Fig. S1 All windows used in *F_ST_* and PMT analyses. Indicated by Z*F*_ST_, correlation coefficients (*r*) and -log_10_*P*.


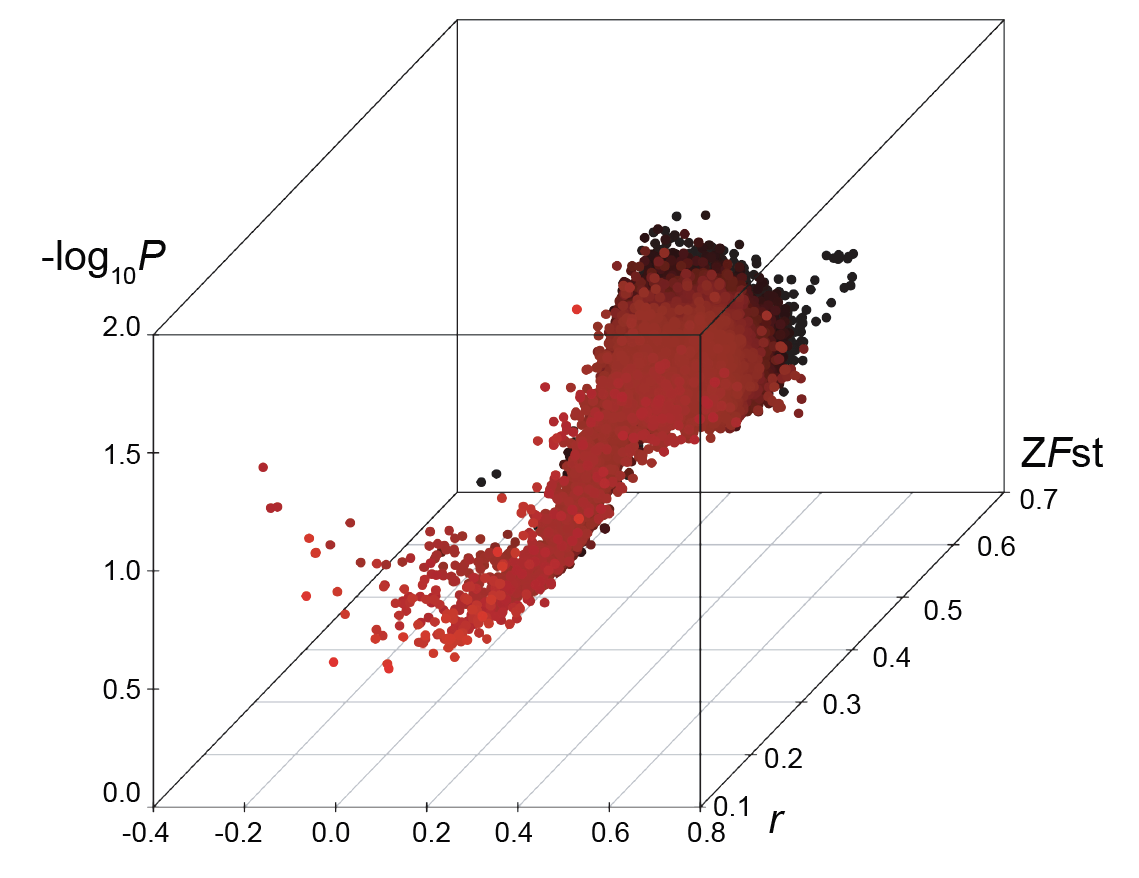


Fig. S2 The phylogenetic tree based on SNPs in open reading frame region of *PTPA* gene.


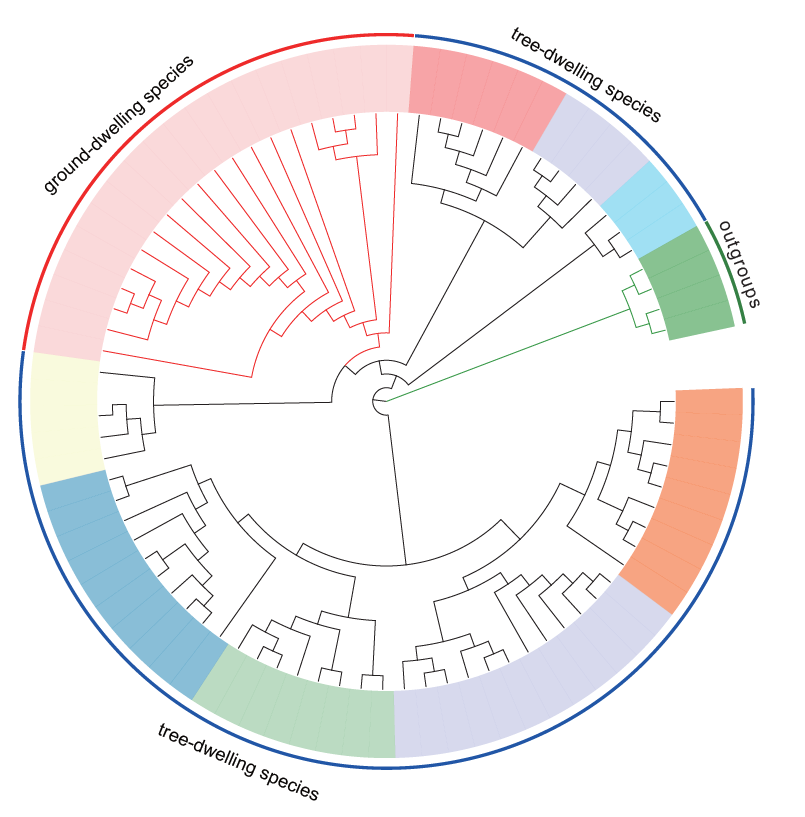


Fig. S3 Protein structure prediction of *PTPA* gene by AlphaFold2. A non-synonymous substitution 13 (E13G) result in a fork at the N-terminal is red.


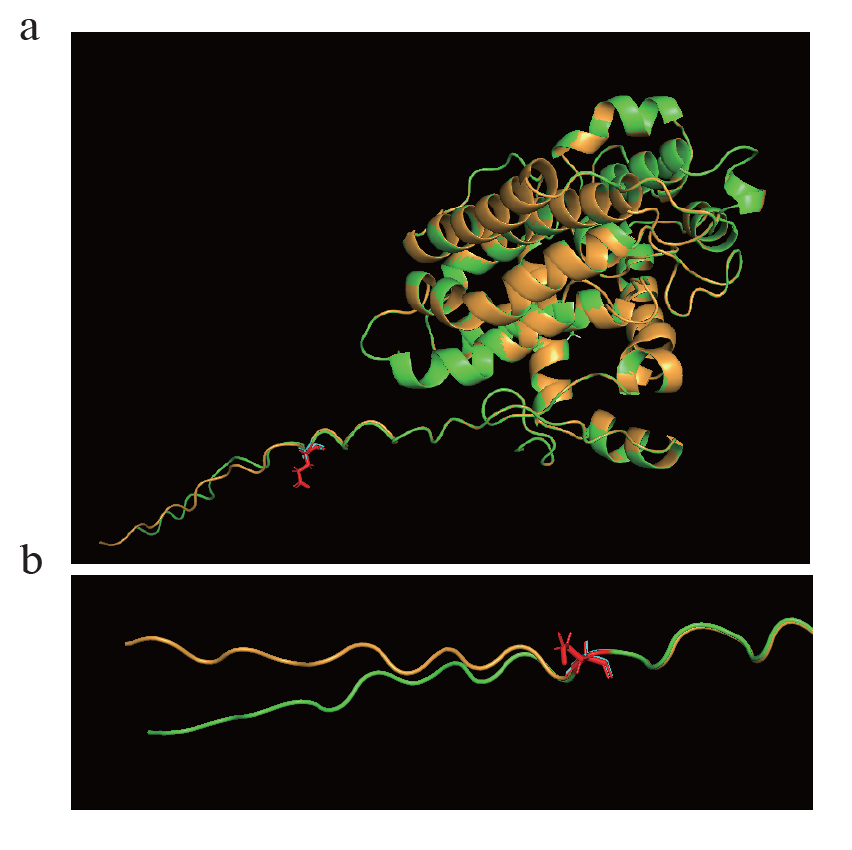

Supplement: evad166_Supplementary_Data [file evad166_supplementary_data.zip › Supplement.docx]
